# College Student’s Academic Help-Seeking Behavior: A Systematic Literature Review

**DOI:** 10.3390/bs13080637

**Published:** 2023-07-31

**Authors:** Ruihua Li, Norlizah Che Hassan, Norzihani Saharuddin

**Affiliations:** Faculty of Educational Studies, Universiti Putra Malaysia, Serdang 43400, Selangor, Malaysia; gs59876@student.upm.edu.my (R.L.); norzihani@upm.edu.my (N.S.)

**Keywords:** college students, academic help-seeking, academic performance, systematic literature review, ATLAS.ti 22

## Abstract

Seeking academic help has a positive impact on students’ ability to handle challenges, leading to improved academic success. As the academic landscape becomes more competitive, the importance of students seeking and using academic support is widely recognized for enhancing their learning experience and achievements. The main objective of this study is to review the prior literature that has examined the academic support provided to college students, addressing the knowledge and methods required in an academic help-seeking process. Based on a systematic literature review, this study’s data were gathered from a review of 55 documents from the 11 years between 2012 and 2022. The literature was then individually analyzed using the ATLAS.ti 22 programs. The analysis shows five central themes: (1) Defining student help-seeking; (2) Academic help-seeking and academic performance; (3) Resources of academic help-seeking; (4) Factors of academic help-seeking; (5) Academic Help Seeking Online. This study also identifies potential new directions for future research that could be useful to school administrators in developing policies to assist students with help-seeking behavior, which could have significant implications for the theoretical development and practical guidance of student help-seeking behavior.

## 1. Introduction

As higher education entered the era of massification in many nations during the 20th century, an increasing number of individuals have been able to attend college [1]. Meanwhile, university assignments have become increasingly complex and challenging. At college, students face a variety of academic obstacles [2,3,4,5]. Learning is hardly ever accomplished alone. Therefore, students should seek help from their teachers or peers [6,7,8], the school’s counseling service [9], or the Internet [10]. The ability to actively seek academic assistance to promote academic success is one of the most important study skills college students must possess. If students struggle with completing their projects, they may want educational support to figure out the issue or challenge themselves [11]. People may experience this scenario psychologically in many ways, engage in various thought processes, and adopt various behaviors. From a learning adjustment perspective, academic help-seeking (AHS) is often considered to be a more important and effective self-regulation strategy [12,13,14]. Obtaining academic help is also an essential self-regulated learning strategy for college students, which plays a significant role in their academic careers [15,16,17,18]. AHS involves seeking support from individuals and other sources to help students do well in the academic environment [10,19].

Since the 1980s, numerous studies have been published that investigate the definition and structure of AHS as a construct [20,21] and relate it to other key variables in the education disciplines [22,23,24]. Nonetheless, due to its social aspects, AHS exhibits diverse individual traits. Its complexity manifests itself on various levels, including the factors that impact AHS, the performance of AHS, and its effects. Interest in this topic has developed during the past decade [25]. College students’ academic help-seeking behavior is a complex phenomenon that has garnered significant research attention. However, there is a need for a comprehensive understanding of this behavior, including its characteristics, trends, and various aspects related to its definition, relationship with academic performance, available resources, influential factors, and the impact of online platforms. Additionally, a systematic evaluation can synthesize existing research on college students’ academic help-seeking behavior and provide a holistic view of the topic. And hence, this study examines a selection of the literature about college students’ academic help-seeking behavior and focuses on answering the following questions:

RQ1: What are the characteristics and trends of the articles related to college students’ academic help-seeking behavior published between 2012 and 2022, considering the country of origin, journal sources, and publication timeline?

RQ2: What insights can be gained regarding college students’ academic help-seeking behavior, including its definition, relationship with academic performance, available resources, influential factors, and the impact of online platforms?

RQ1 focuses on identifying the characteristics and trends of articles published between 2012 and 2022, considering factors such as the country of origin, journal sources, and publication timeline. This analysis will provide insights into the research landscape, highlighting key contributors and temporal patterns in the field. RQ2 delves into the various aspects of college students’ academic help-seeking behavior, including its definition, relationship with academic performance, available resources, influential factors, and the impact of online platforms. By examining these dimensions, the study aims to uncover valuable insights that can inform the development of effective interventions and support services for college students. Furthermore, this systematic review will identify research gaps and propose future research directions. It will shed light on common barriers and facilitators affecting students’ engagement in academic help-seeking, ultimately contributing to the advancement of knowledge in this field.

## 2. Background Literature

### 2.1. Academic Help-Seeking Behaviors

“Help-seeking” is a structured and interactive social behavior that has been found to have a positive correlation with academic achievement among students [26]. In one of the earlier studies, Karabenick and Knapp [14] noted that the distinction between formal and informal academic assistance was made. The formal ones included resources from mentors and schools, while the informal ones included friends, peers, and family. Nelson-Le Gall and Jones [27] found two types of student help-seeking behavior observed: one in which students were more independent in their use of AHS behavior and another in which they were more dependent. When seeking executive assistance, students wish to receive answers to their questions without any effort on their part. When students wish to improve their learning and problem-solving abilities, they seek instrumental assistance. It is also viewed as a self-regulatory learning strategy for students to seek academic assistance [28]. Another definition of help-seeking is by Ryan and Pintrich [29], who defined it as the capacity to utilize others or other resources to solve problems when confronted with learning difficulties or challenges and complex situations. Umarani [30] reminds us that seeking academic help is a learning strategy that can effectively facilitate student learning and ultimately benefit students’ academic performance. About the help-seeking process, Karabenick and Dembo [31] outlined eight steps: (1) decide whether a problem exists; (2) decide whether assistance is required or desired; (3) determine whether to request assistance; (4) choose the type of help you want (executive or instrumental); (5) choose the person you want to seek for help; (6) ask for help; (7) get help; (8) process the help you got. These steps must be carried out in an effective manner, which calls for cognitive, social, and emotional competencies that are instructible to students who might be lacking in these abilities.

### 2.2. Relationship between Academic Help-Seeking and Academic Success

For many years, researchers have researched the relationship between AHS and academic performance, with most studies indicating that academic help-seeking behavior has a favorable effect on academic success. Micari and Calkins [32] showed that teachers who are receptive to students’ requests for assistance will receive increasingly higher grades. In other words, students will have a higher GPA and eventually succeed in college if they ask their teachers for assistance more frequently. One study by Umarani [30] found that students with academic difficulties who actively seek academic assistance can improve their academic performance. In general, students who refuse academic support perform worse in school than those who regularly interact with their teachers. Another study by Algharaibeh [33] offered an analysis of the various sources of academic help, including the fact that formal sources are typically school teachers and academic service centers provided by the school, whereas informal sources are typically parents, peers, classmates, etc.

Whether formal or informal, help-seeking can improve academic performance, encourage positive learning, and increase students’ sense of self-efficacy. Previous research on academic help-seeking has demonstrated that seeking assistance from official sources (e.g., teachers and academic service centers) or informal sources (e.g., peers and family) promotes positive learning trends, increased self-efficacy, and enhanced academic performance [19]. While students’ academic help-seeking behavior is very negative in traditional learning environments, primarily because students believe that asking for academic help in public implies that they are not capable of learning and because they believe it has an impact on their self-esteem [34].

## 3. Materials and Methodology

This study employs a literature review approach to fulfill its objective of identifying the key aspects of a subject or topic through analysis of previous research to identify research gaps. Specifically, the study is a thematic review of relevant literature. By allowing for thematic grouping, a thematic literature review enables researchers to showcase the specific topics that are most relevant to their research. The present study employs a thematic literature review as its primary analytical technique, which involves searching for and analyzing relevant data obtained from databases. This approach has been utilized by numerous previous studies [35,36,37,38]. The Preferred Reporting Items for Systematic Reviews and Meta-Analysis (PRISMA) were used to manage the literature search [39].

### 3.1. Databases and Search Terms

Multiple strategies were employed to obtain a wide range of related studies within the scope of this SLR [40]. We use the electronic databases Web of Science (WOS) and Scopus, ERIC (Education Resource Information Center), PsycINFO (EBSCO), and ScienceDirect to search the included articles. For each characteristic (help-seeking, college students), we used multiple terms to enhance our ability to find as many relevant articles as possible. For the characteristic of help-seeking, we use terms: Online academic help-seeking OR OAHS OR academic help-seeking OR AHS OR academic help-seeking’ OR ‘academic advising’ OR ‘academic advice’ OR ‘intrusive advising’ OR ‘support service’ OR ‘support services’ OR ‘academic support’ OR “help seeking” OR “help-seeking” OR “Help-seeking behavior” “Help-seeking intentions” OR “Learning strategies” OR “Question asking” OR “self-regulated learning”. For the characteristic of college students, we used the terms: “university” OR “college” OR “academy” OR “higher education” OR ‘university student’ OR ‘university students’ OR ‘college student’ OR ‘college students’. Within each category, keywords were joined with OR, and terms were joined with AND between each category A string was adopted in five databases (Table 1). To ensure the assessment was comprehensive, we also used backward and forward snowball search techniques [41]. The five focal databases were then mined for a total of 1839 articles.

### 3.2. Inclusion/Exclusion Criteria

All searches were carried out in June 2023. Papers were included for consideration using the following criteria: (1) publication dates range from 2012–2022; (2) the following keywords must be included: college students, academic help-seeking, help-seeking behavior, and undergraduate students; (3) the language used in the article is English; (4) quantitative, qualitative, and mixed methods studies are included to consider this research topic in different dimensions. The chosen period for this study is 2012–2022. This selection is based on several factors. Firstly, during this timeframe, online resources were widely used, allowing us to examine the impact of technology integration on college students’ academic help-seeking behavior. Secondly, focusing on the years 2012–2022 ensures access to recent and relevant literature, enabling us to capture the latest trends and advancements in understanding this behavior. Lastly, a narrower time frame allows for a thorough review of the literature, enhancing our understanding of college students’ academic help-seeking behavior.

Criteria for exclusion: (1). Articles focusing on individuals outside the college student population, such as K12 students, adults, elderly individuals, disabled individuals, and non-college groups, will be excluded from the review. (2). Articles that primarily focus on help-seeking behaviors related to physiological concerns, psychological issues, or mental health conditions, rather than academic help-seeking, will be excluded. (3). Articles that do not directly address the research question and are not related to the definition of academic help-seeking, resources for academic help-seeking, influencing channels, and factors of online academic help-seeking will be excluded. (4). Non-empirical articles, such as literature reviews, theoretical papers, opinion pieces, and editorials, will be excluded from the review. Only empirical studies reporting original research findings will be considered. (5). Articles written in languages other than English will be excluded from the review, as the analysis will focus on English-language publications. (6). Papers that are not available in full text.

### 3.3. Selection of Articles and Descriptive Overview

**Figure 1:**
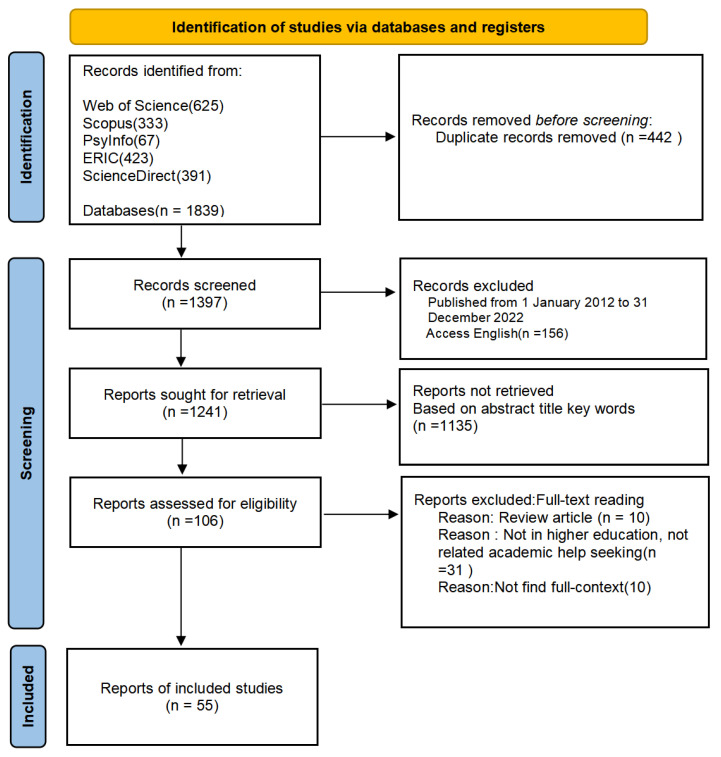
PRISMA flow diagram of the research process.

All searches were carried out in June 2023. Web of Science (WOS), Scopus, ERIC (Education Resource Information Center), PsycINFO (EBSCO), and ScienceDirect were used to conduct the literature search for this study, and they returned 1839 results. In the first step, 1839 articles were stored in Endnote X9, and 442 duplicates were removed. We went on to remove 156 documents based on the range of time and language. In the second phase, two independent investigators screened the articles against the eligibility criteria based on the title, abstract, and keywords, 1135 articles were removed. After a full-text examination, 55 articles published between 2012 and 2022 were selected for qualitative synthesis. Figure 1 summarizes the three phases of the process of choosing references for analysis and prior studies (identification, screening, and inclusion).

### 3.4. Quality Assessment of Included Studies

To assess the quality of each study included in the review, we utilized Crowe’s critical appraisal tool (CCAT). The suitability of this tool for the study was justified by its capability to accommodate various study designs, such as quantitative, qualitative, and mixed-methods studies. Moreover, CCAT is highly reliable [42,43]. The CCAT consists of eight category items, which are Preliminaries, Introduction, Design, Sampling, Data Collection, Ethical Matters, Results, and Discussion. Each category item is scored on a five-point scale, resulting in a maximum aggregate score of 40. (See Appendix A Table A1) The CCAT User Guide provides detailed explanations and references for how each category item can be scored [44] (See Supplementary Materials). The CCAT was utilized by the first author for each study, and the second author independently applied it to more than half of the research. Any discrepancies that arose were resolved through ongoing discussions. The characteristics and CCAT scores for all 55 studies are presented in Table A2 (See Appendix B Table A2).

### 3.5. Approach to Analysis and Synthesis

For analysis, 55 articles in total were uploaded to ATLAS.ti 22. And using ATLAS.ti, 22 were used for the literature review analysis introduced by Zairul [45]. Each article was categorized by the author, journal name, journal number, publisher, and year of publication. Quantitative and qualitative findings are presented in this paper. The quantitative section focuses primarily on the regional, journal, and national distribution of academic research articles. In the section on qualitative data analysis, the primary method employed was thematic analysis, which was used to classify and summarize the articles and ultimately construct the framework while ensuring that the framework and data were linked [46]. This method involves coding, categorizing, and refining themes extracted from raw data [47]. The present research follows the 6-step framework outlined by Braun and Clarke [48]. The steps follow as (1) becoming familiar with the data, (2) generating codes, (3) identifying themes, (4) reviewing themes, (5) defining themes, and (6) explaining themes.

We began the thematic analysis by immersing ourselves in the data and gaining familiarity with the content of the articles about the research question. In the subsequent two steps, we initially assigned codes to the articles based on general themes, using an inductive approach that allowed themes to emerge from specific observations in the empirical studies. This involved focusing on key aspects of the articles, such as the author, year of publication, the country, the purpose of the study, study design, and study conclusions, and extracting recurring subject terms, such as AHS, types of AHS behavior, and online AHS behavior. In the fourth step, we reviewed all established themes and sorted out any overlaps or strongly interrelated themes. In the fifth step, we merged and defined all the shortlisted themes, continuously revising them until all sub-themes were grouped under the main theme. In the final step, we refined and defined the themes further, with the first two authors reaching a consensus on each theme through discussion and consideration of its connection to the research question. If there were any disagreements, a third author was consulted. After reviewing and validating the initial codes, we proceeded to identify and refine the themes. The resulting set of themes and their analysis are presented in the following section. Through an interactive process, we categorized the initial codes into broader subjects and had discussions among the authors, which led to the identification of five themes: (1) Defining student help-seeking, (2) Academic help-seeking and academic performance, (3) Resources for academic help-seeking, (4) Factors influencing academic help-seeking, and (5) Online academic help-seeking.

## 4. Results

Two categories of results are presented: quantitative and qualitative. The quantitative portion of the study will address question 1, whereas the qualitative portion will address question 2. Despite an expanding body of research on students’ AHS behaviors, there are currently no review papers that provide a comprehensive study of students’ AHS behaviors as well as a framework for future research.

**Figure 2:**
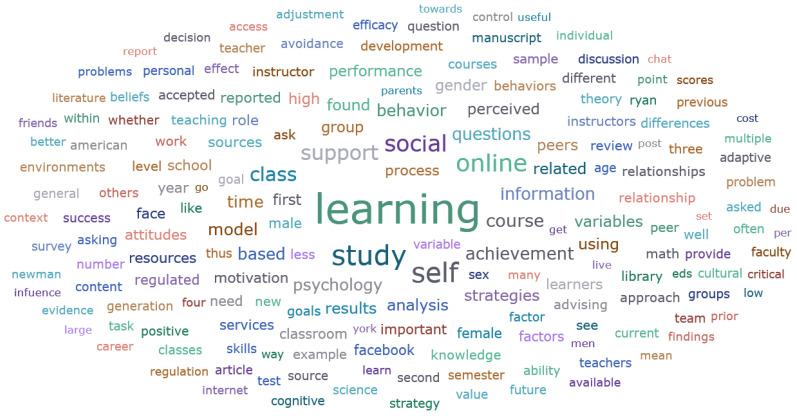
Word cloud map generated in 55 documents.

**Figure 3:**
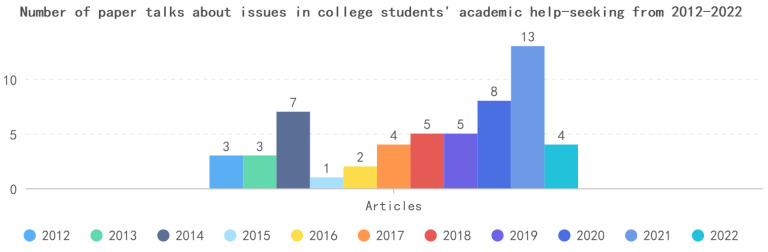
Several papers about college students’ academic help-seeking.

The word cloud was created in ATLAS.ti 22 software, after adding the keywords “help”, “seeking”, “academic”, and some numbers and special characters to the stop word list. “The results of the word cloud revealed that the most prominent concepts were learning, study, online, support, social, class, and information. (See Figure 2). As the number of journal articles on academic research has increased in recent years, from three articles in 2012 to seven articles in 2014 to thirteen articles in 2021 (See Figure 3). Importantly, this analysis does not currently exclude any limitations, and the literature being analyzed is the literature chosen for the research question.

### 4.1. Quantitative Results

According to the findings, college students’ AHS behaviors were primarily published in these journals. According to the list shown in Table 2: The top three journals for AHS behavior among college students are Frontiers in Education, Internet and Higher Education, and Journal of Academic Librarianship. The journals International Journal of Educational Technology in Higher education, Journal of College Student Development, and TechTrends published two articles in the last 10 years. The remaining 39 journals published one article on a related topic in the last 10 years. In the process of searching the literature, we found that some of the articles are about academic help-seeking behavior, but the keywords of the articles are self-regulated, learning strategy, asking questions, etc., which are also related to academic help-seeking. Therefore, in the process of searching the literature, we found that if we expand the search terms, we will find that the literature on academic help-seeking behavior has shown a gradual increase in recent years.

**Figure 4:**
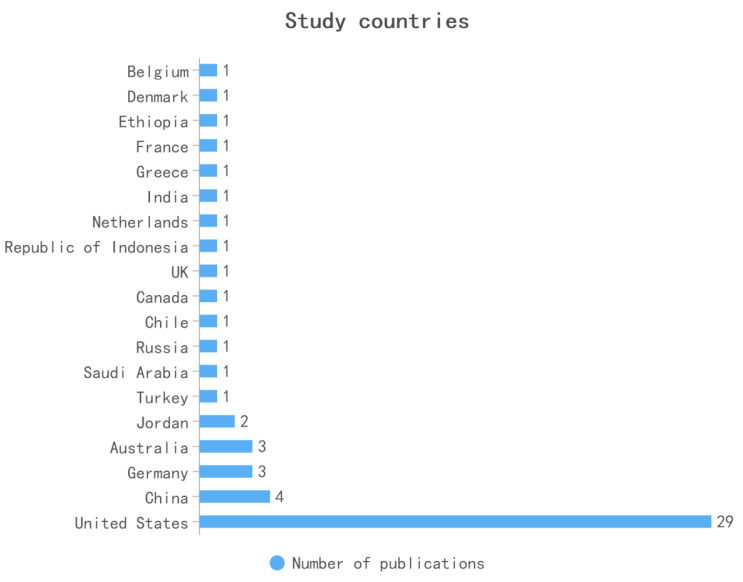
Study countries.

The United States, China, Germany, and Australia are the leading regions for academic research on university counts, with the United States publishing the most articles on the AHS behavior of college students over the past decade. One of the papers investigates how the emergence of higher education’s cultural mismatch influences the academic assistance-seeking behavior of first-generation college students [49]. Payakachat et al. [50] and Finney et al. [51] are more focused on College students’ behavior in seeking academic assistance. In Germany, to better understand self-reported help-seeking strategies, Zander and Hoehne [52] analyzed autonomy-oriented, dependency-oriented, and help-seeking avoidance in undergraduate computing and pedagogy programs. Schlusche et al. [53] used a survey to investigate the impact of social resources on the relationship between AHS behavior and the academic performance of college students in the lower division. Consideration was given to the regional distribution of the Institute (Figure 4). AHS behavior among college students has long been studied in several countries. There has been a lot of recent research on college students in Asian nations, including Taiwan and mainland China, who seek academic assistance. Nonetheless, the AHS behavior of Asian college students demands additional analysis.

In conclusion, this section responds to RQ1: What are the specific characteristics and trends of the articles published between 2012 and 2022? The reviewed articles discussed AHS behaviors and processes of college students, the most recent of which is more specific about demographics (gender age, and attitudes) that affect students’ behaviors of AHS, but some related research is still less when compared to the K12 study. Meanwhile, almost half of the research is about American students, and other countries are just coming into focus on this top. Especially in China, there are few studies about the AHS behaviors of college students. But it is worth noting that more and more countries are joining and researching college students’ AHS behavior, and the research is increasingly focused and refined.

### 4.2. Qualitative Results

**Figure 5:**
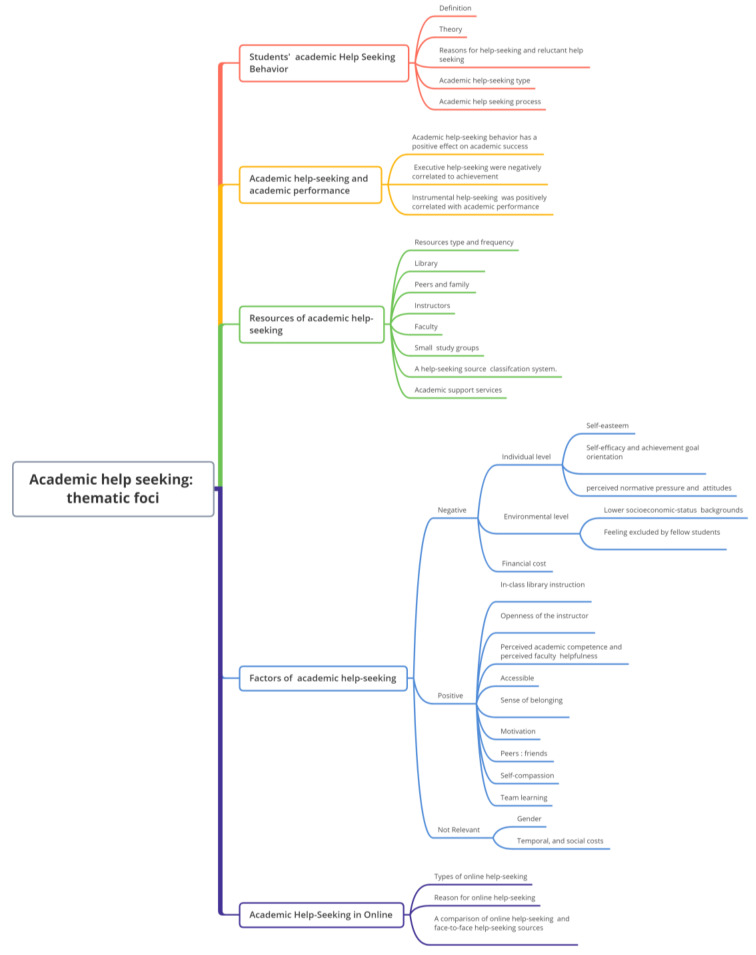
Key themes and subthemes in the literature on academic help-seeking.

To respond to the second research query, the literature was further analyzed in the qualitative analysis section. We carefully read and coded 55 articles on the behavior of college students at AHS. Coding was not completed in one sitting. The initial codes must be merged and classified to form the themes. Some codes that are rarely used or cannot be incorporated into the current theme will be eliminated, primarily because we are concentrating on universal elements. Firstly, the definition and categorization of AHS behavior as a concept and behavior of college students. Secondly, various viewpoints are used to analyze the facilitating and impeding factors of AHS behavior, as well as its influencing factors. Thirdly, the use of on-campus and off-campus resources, conventional AHS, and online AHS behavior about AHS behavior. After that, it is discussed how AHS behavior fosters academic progress, and then AHS behavior in the Internet era is examined (See Figure 5). Future research directions can be deduced from the existing research and conceptual framework; this section will be developed specifically in the discussion section.

#### 4.2.1. Theme 1: Defining Student Help-Seeking

Topic 1 focuses on the definition and theories related to students’ AHS behavior, as well as the reasons why students seek help and the circumstances under which they refuse to seek help. It also describes the classification of student help-seeking behavior and concludes with recent advances in student help-seeking behavior. Ames and Lau [54] defined AHS as a method of locating and utilizing additional resources for one’s success.”. When an individual recognizes that they cannot overcome their problems on their own, help-seeking behavior develops. In general, the help-seeking process entails acknowledging the need for assistance, locating potential people who can assist, articulating the problem that needs to be solved, and evaluating the outcome of the help-seeking process. Likewise, Almaghaslah and Alsayari [9] hold the view that the behavioral strategy of actively seeking academic assistance is through social interaction. Beisler and Medaille [55] explained the students’ perceptions of academic help as an effective problem-solving strategy. According to these studies, asking for AHS is a good learning strategy. Students use AHS behaviors to address their academic challenges and difficulties through their efforts and ultimately to achieve academic success because they are the main subjects of learning in the university setting.

Some authors view AHS as a self-regulation strategy employed by students [20,26]. Unlike other cognitive strategies, this AHS combines cognitive and social integration skills. The first step in a student’s process of seeking help is becoming aware of the need for it. Therefore, when a student seeks assistance, a series of choices are made. These choices could be but are not restricted to becoming aware of the issue and challenge; choosing to seek assistance; choosing from whom to seek assistance; choosing when to seek assistance; selecting the form of assistance to seek [51]. Recently, Payne utilized Yosso’s community cultural wealth framework, understanding how the academic help-seeking behavior of first-generation college students can lead to success in the field of post-secondary education through the accumulation of their cultural capital. Meanwhile, they conducted a systematic study of first-generation college students’ academic help-seeking behavior and concluded that academic help-seeking provides students with a source of power over their family’s cultural capital as well as the school’s cultural capital collision [56].

Reeves and Sperling [57] claimed that a student’s AHS behaviors are significant for predicting performance and assisting students in overcoming academic obstacles by accepting associate instruction from a school. Similarly, Almaghaslah and Alsayari [9] asserted additional motivation to complete academic courses and improve academic performance improves students’ help-seeking behavior in learning. Academic help-seeking behavior can be extremely beneficial to a student’s academic success, but many students do not use it successfully; for example, Schworm and Gruber [58] used a survey to find that college students are reluctant to seek academic assistance in traditional classroom settings. This view is explained by Mahasneh et al. [59], who wrote that this effect may be influenced by the absence of necessary background information and the perception of danger associated with seeking assistance.

A broader perspective has been adopted by Almaghaslah and Alsayari [9] who argued that in the definition of academics, formal academic help-seeking is generally considered to be seeking help from teachers in the classroom and formal academic institutions in school, whereas informal AHS is primarily seeking help from classmates, friends, peers, or family members. Help-seeking comes in two forms [14,60]: The first type of help-seeking behavior is rapid help-seeking, also known as executive help-seeking, which is primarily characterized by seeking the best solution directly from teachers or peers, without thinking. The second type, referred to as slow help-seeking behavior or instrumental help-seeking, is primarily characterized by the assistance of others who can eventually complete the task on their own [52]. Beisler and Medaille [55] described when a student requests quick or executive help, they are searching for an immediate fix and are not concerned with significantly contributing to the help-seeking process. However, students want to be able to develop their learning and problem-solving abilities during the AHS process when the type of help sought is instrumental. There is also a classification by Reeves and Sperling [57], who identified two orientations of student help-seeking behavior, adaptive, and avoidant. Pupils who are adaptive help-seekers are more capable of engaging in positive academic help-seeking behaviors, whereas those with avoidant orientations are more likely to rely on their strengths to solve problems. The study of students’ academic help-seeking behaviors can now be viewed in a new light thanks to decision-inspired methods. In addition, several research techniques should be used to investigate the types of resources students use to seek academic assistance in their actual behavior [61].

Current research on students’ help-seeking behavior has concentrated primarily on academic perspectives instead of investigating how students recognize and perceive their help-seeking behavior. Theories of learned help-seeking behavior are more diverse, ranging from a psychological perspective that views it as a learning strategy and dissects the process of help-seeking behaviors to a cultural capital perspective that views it as a type of cultural capital that students can use. Different classifications of help-seeking behavior are made from various perspectives, providing us with a more thorough comprehension of help-seeking behavior.

#### 4.2.2. Theme 2: Academic Help-Seeking and Academic Performance

Active help-seeking in academics is a prosocial, structured, and interactive behavior that promotes students’ academic growth. And in the fields of education and psychology, help-seeking has been one of the key research themes [33]. The link between academic assistance and academic achievement has been studied for many years, with most studies concluding that academic help-seeking behavior has a positive effect on academic success. Karabenick and Knapp [14] asserted that a student’s academic performance can be improved by seeking academic assistance from peers and teachers. Schworm and Berndt [62] found that one of the most crucial study skills for college students to have to succeed in their studies is the ability to ask for AHS which is supported by Payakachat, Gubbins, Ragland, Norman, Flowers, Stowe, DeHart, Pace, and Hastings [50]. And, Umarani [30] reminds us AHS is a learning strategy that can effectively facilitate student learning and ultimately benefit students’ academic performance. Micari and Calkins [32] showed that teachers who are receptive to students’ requests for assistance will receive increasingly higher grades. In other words, students will have a higher GPA and eventually succeed in college if they ask their teachers for assistance more frequently.

One study by Umarani [30] examined that seeking academic assistance is an academic process learning strategy for students. And, students with academic difficulties who actively seek academic assistance can improve their academic performance. In general, students who refuse academic support perform worse in school than those who regularly interact with their teachers. Another study by Algharaibeh [33] offers an analysis of the various sources of academic assistance, including the fact that formal sources are typically school teachers and academic service centers provided by the school, whereas informal sources are typically parents, peers, classmates, etc. Help-seeking, whether formal or informal, can improve academic performance, encourage positive learning, and increase students’ sense of self-efficacy. Additionally, there are studies in which researchers have looked at how academic achievement and help-seeking fare across disciplines. In a cross-sectional study conducted by Rini and Wijanarko [63], it was shown that the Nursing Science Research Project at the Muhammad Foundation in Bali found a positive correlation between seeking academic assistance and student achievement. Sun et al. [64] also found that there was a significant positive correlation between students’ self-efficacy in learning math, their utilization of help-seeking strategies, and academic achievement in both pre-and in-class learning settings. Zheng and Zhang [65] contended the use of peer learning and help-seeking positively affected the performance of first- and second-year students in the flipped classroom.

In the realm of educational research, help-seeking behaviors among students have long been acknowledged as crucial determinants of academic development. Nevertheless, not all help-seeking behaviors are created equal. In earlier research, the terms expedient help-seeking and adaptive help-seeking were distinguished [20,66]. Expedient help-seeking typically involves students looking for shortcuts, often expecting others to complete tasks for them or directly asking for solutions without seeking genuine understanding. Such behaviors can be counterproductive, sometimes resulting in poorer academic outcomes and heightened levels of student anxiety. In contrast, adaptive help-seeking behavior, also known as instrumental help-seeking behavior, occurs when students seek assistance by considering other people or resources and eventually solve the problem on their own [21,67].

#### 4.2.3. Theme 3: Resources of Academic Help-Seeking

Early studies on college students’ AHS behavior concentrated on patterns of AHS behavior and the variables affecting that behavior. The use of specific academic resources, such as academic service centers in libraries and schools and online help-seeking within the scope of distance education, has emerged in more recent studies of the academic literature [9]. The following section discusses the different types of academic assistance resources, such as peers, classmates, friends, teachers, libraries, academic service centers, and the Internet. With varying regularity and efficacy, students use various kinds of academic support tools. Effectiveness, timeliness, cost, accessibility, and for students, user-friendliness is the most important factor to consider when selecting academic support resources [9].

A mixed study by Beisler and Medaille [55] described eighty students who used drawings to describe their AHS behavior; the results revealed that 59% of the students sought assistance from a peer or family member, followed by their tutor and the school’s writing center. This view is supported by other scholars who concur that when students encounter academic difficulties, they typically seek assistance from their peers. Mahasneh, Sowan, and Nassar [59] found that peers are students’ first choice when looking for academic assistance. Moreover, more than forty percent of students who encounter academic difficulties attempt to find solutions on their own. Likewise, Almaghaslah and Alsayari [9] hold the view that peers, online course portals, and online educational resources are the three most popular types of resources used by students following an academic call for assistance. In the context of higher education, university instructors do need to understand that one of the components of their student’s academic success is their support of them [68]. The behavior of the instructor in the classroom has a direct impact on the effectiveness of the student’s lessons, their attitudes toward learning methods, and ultimately the quality of their learning [32]. In a similar vein, Thomas et al. [69] in their article noted that to succeed academically, students first ask for assistance from their teachers and peers.

When the help-seeking scenario arrived in the school setting, scholars conducted the following research. Giblin, Stefaniak, Eckhoff, and Luo [61] conducted a similar experiment at a university, and the results revealed distinct manifestations of students’ AHS behavior in and out of the classroom. In the classroom, 43% of students chose their classmates for academic assistance, while 17% chose their notes; however, outside the classroom, 39% chose online resources, and 28% chose classmates or friends. Less frequently did students utilize textbooks, class notes, and their teachers’ instruction. Moreover, the study revealed that students formed study groups and utilized multiple websites to achieve their AHS behavior. Another study showed that email was the most popular resource for academic assistance before and after class, whereas discussion and office hours were the least popular [57].

Typically, students select their advisor rather than the school’s academic services as the official source of assistance [14]. Academic services are faced with a new challenge because the majority of undergraduate students do not know how to ask for assistance. Elias et al. [70] describe the three main aims that most of these services have in common: (1) educating students about online academic support resources; (2) encouragement of students to seek academic assistance; (3) helping learners to use self-directed learning strategies. The library is also underutilized by students. But, most students are unaware of the library’s resources and the assistance offered by the library staff. A study conducted by Beisler and Medaille [55] indicated that in-class library instruction sessions do seem to have a positive impact on students; however, students do not connect their different research needs with possible library assistance. Wirtz et al. [71] concluded four new patterns in students’ behavior when looking for assistance: Students AHS will have access to a variety of resources, but the frequency of their use is undesirable; (2) The utility of resources is not the only factor motivating student use of them; (3) The ranking of academic assistance resources by students is primarily based on the availability of resources; (4) The time and location of access to resources explain why students seek help, the more convenient a resource is perceived to be, the more likely a student is to use it.

We can draw the following conclusion from the research: when it comes to academic support, students use resources in various ways. The main consideration for students is how convenient the resource is. In other words, students are more likely to select the most convenient resource for them than the most useful one. Therefore, future research should delve deeper into the determinants of student resource selection. In the field of higher education, academic support resources should be made more accessible so that students can more easily seek assistance.

#### 4.2.4. Theme 4: Factors of Academic Help-Seeking

An extensive and expanding body of literature has investigated what factors influence students’ AHS behaviors. Some of these factors can help or hinder students’ help-seeking behavior, while others may be found to not affect students’ AHS after the study. The elements that affect students’ AHS are specifically described below.

Many students believe that seeking academic assistance from others reveals their academic deficiencies, which can negatively impact their self-esteem. The desire of students to seek assistance will decrease if they believe that doing so will bring down their self-esteem [32]. Zander and Hoehne [52] have been able to show that it can lessen students’ behavior toward seeking assistance if they experience exclusion by fellow students. AHS behavior can be reduced by ambivalence and the perception of academic help-seeking threats [50].

According to one study, students were more likely to see the threatening aspect of asking for academic help in person [57]. The main cause of students’ perceived help-seeking threats is low self-esteem, which is brought on by reluctance to admit their failings. Additionally, the learning environment and types of resources available for help-seeking influence students’ perceptions of help-seeking threats [28]. A student may choose not to ask for help for numerous explanations, including the nature of the difficulty they are facing, their study habits and tendencies, their relationship with their preferred potential helper, and the particular circumstances surrounding the request for help at the time [55]. Similarly, Schworm and Gruber [58] also mentioned that students may refuse assistance due to a lack of information or the perceived threat of asking for assistance. Thomas and Tagler [72] in their study used the Reasoned Action Model (RAM) to investigate the determinants of students’ intentions to utilize university-based sources of academic support. They found that perceived normative pressure and attitudes accounted for a considerable amount of variability in intentions to seek help.

Additionally, gender has distinct influences on students’ help-seeking behaviors [73]. Dunn et al. [74] found that as individuals grew older, their tendency to seek help decreased. Furthermore, in Calarco’s [75] study, socio-economic status also influences students’ academic help-seeking behavior; in general, students from lower socio-economic backgrounds believe teachers will respond negatively if they ask for assistance, whereas students from middle-class families do not hold this viewpoint. Commenting on avoiding seeking help, Mahasneh, Sowan and Nassar [59] argued: to begin with, asking for assistance is a dependent learning strategy, so students may avoid doing so when putting the idea of independent and autonomous learning into practice. The second is that students might interpret asking for assistance as an indication of incompetence. Third, asking for academic help is a socially interactive behavior, so how the student perceives the academic environment around him, or she may have an impact on how the student asks for assistance. There is also a claim that if a student feels uncomfortable in the classroom when interacting with peers or the teacher, this may deter them from asking for assistance.

Significant amounts of the literature have been published on students seeking academic assistance. Several encouraging influences on students’ willingness to ask for help were uncovered by these studies. For example, Beisler and Medaille [55] uncovered that direct academic instruction in the classroom encourages students to seek out academic help-seeking. Micari and Calkins [32] showed that positive attitudes toward students’ help-seeking behaviors will result in more academic help-seeking behaviors at the course’s conclusion.

Additionally, if students are given more incentives for helping, their behavior of asking for assistance will support their academic success, and they will be more attentive to the subject matter and engaged in class discussions [58]. Students’ instrumental help-seeking behavior is influenced, as expected, by a collaborative approach to course learning [62]. Thus far, Dunn, Rakes, and Rakes [74] demonstrated that academic self-discipline and thinking critically positively influence academic help-seeking behaviors, and as academic self-discipline and critical thinking increase, so do academic help-seeking behaviors.

And, in a study carried out by Payakachat, Gubbins, Ragland, Norman, Flowers, Stowe, DeHart, Pace, and Hastings [50], it was shown that how students act when they need help in school can be affected by how smart they think they are and how helpful their teachers are. Brouwer and Engels [76] greater emphasis was placed on examining the impact of peers on students’ tendencies to seek help, revealing that they were more inclined to seek assistance from a friend. Opdecam et al. [77] indicate that students who favored team learning had lower ability levels but higher intrinsic motivation, less control over their learning beliefs, increased help-seeking behavior, and a greater willingness to share knowledge with peers. In addition, the similarity of academic achievement among friends contributed to the occurrence of help-seeking behaviors and ultimately led to the formation of the student’s academic help-seeking network. As noted by Won et al. [78], even when considering the student’s motivation to seek help, the student’s perceived sense of belonging predisposes the student to adaptive academic help-seeking behavior. In the meantime, self-efficacy for self-regulated learning positively predicted adaptive help-seeking strategies as well. When faced with challenges or difficulties in their academic work, college students are more likely to seek assistance if they have greater confidence in their ability to self-regulate their studies. Long and Neff [79] noted that self-compassion indirectly promotes help-seeking by reducing the fear of positive evaluation. Additionally, self-compassion directly encourages help-seeking due to the focus on personal well-being.

The impact of gender on academic help-seeking is currently viewed in different ways. As noted by Zander and Hoehne [52], women are typically more proactive than men in seeking academic assistance. While in another two studies, researchers found gender is not a variable that impacts help-seeking [80]. In another experimental research, Miranda Lery Santos et al. [81] compare the economic, time, and social costs of help-seeking to the expected benefits, and found participants were more likely to seek help when there was no economic cost to help, but were not as sensitive to the time cost and social cost parameters.

Overall, there seems to be some evidence to indicate numerous factors affect how students behave when they need academic assistance. For instance, while some pursuits of academic excellence and self-affirmation can encourage constructive help-seeking behaviors, others, such as low self-esteem, uncertainty about issues, and failure-related fear, can have an impact on students’ efforts to get assistance. The school environment and the social environment in which students live can also affect students’ help-seeking behaviors, along with gender and age.

#### 4.2.5. Theme 5: Academic Help Seeking Online

Obtaining AHS via the Internet is a novel method. The following section compares the effectiveness of face-to-face help-seeking, hybrid help-seeking, and online help-seeking. In addition, the role of Facebook and live chat in academic help-seeking behavior is described.

To date, several studies have defined what it means to obtain AHS online. Seeking academic assistance through online platforms or tools, such as search engines, email, instant messengers, and social media, is referred to as seeking academic help online [82]. Students seeking online academic help can receive both formal and informal academic assistance, and doing so is a strategy for succeeding academically [83]. Broadbent and Lodge [84] thought that students primarily turn to the Internet for academic help from peers, friends, and teachers to overcome academic obstacles. In addition, email, forums, social media, and classroom questioning interactions were the most common online resources used to seek academic help.

According to a quantitative study of college students in Taiwan, getting academic assistance online is the new way to learn, and these students are more inclined to use online resources to look up solutions to their academic problems [85]. Students who frequently used Facebook formally and informally for academic support perceived the web as socially interactive, were able to foster a sense of community through online interactions, and were more likely to seek academic assistance online [15]. Another important study by Broadbent and Lodge [84] explained why students like to seek help online. Two reasons cited significantly more frequently by online students as justifications for their preference for live chat are highlighted by thematic analysis. Live chat may be the most popular method for online students to seek assistance. This is primarily because they have greater access to the instructor and can communicate in this manner to facilitate face-to-face interaction and because live chat enables the staff to respond quickly.

When compared to face-to-face contact, Mahasneh, Sowan, and Nassar [59] noted that online learning environments encourage students to use help-seeking techniques more frequently than traditional classroom settings do. However, in a recent quantitative study, Reeves and Sperling [57] investigated that students still plan to rely more on interpersonal rather than technological channels of support, despite the threat. In one well-known web-based survey, Tang [68] reported that Students with greater self-efficacy frequently visit the library and seek academic assistance from afar. In-person rather than online academic assistance is preferred by students who live close to their school. Email remains the most common source of academic assistance for students who live far from school.

As Hayman et al. [86] stated that students use their Facebook confessions in four different ways to support their academic experiences and guide their undergraduate careers: they ask for help on Facebook, give study advice, look for useful information, and control their study habits. Broadbent and Lodge [84] compared opinions of live chat technology used for online academic help in higher education between online and blended learners. Because they feel more cared for by the teaching team through timely chat, online students are more satisfied with live chat and are more likely than blended students to suggest others use this form of assistance. Furthermore, Hao, Wright, Barnes, and Branch [82] investigated computer science majors’ online help-seeking behavior was predicted. For each of the three online help-seeking categories, the biggest predictor was the degree of difficulty of the problem. Learners’ ability level, academic performance, and epistemological beliefs were significant predictors of online search and faculty help-seeking. Barnard et al. [87] conceptualize Self-regulated Learning (SRL) as a complex construct consisting of six dimensions: environment structuring, goal setting, time management, help-seeking, task strategies, and self-evaluation. Vilkova and Shcheglova [88] survey to evaluate the function of SRL dimensions and found that low communication between MOOC students and instructors during the learning process implies that the dimension ‘help-seeking’ is not effective in the MOOC environment.

Taken together, these studies prove the proposition that academic help-seeking online is accepted by most students. Additionally, requesting assistance online provides better and quicker access to elementary teachers, protecting students’ privacy while also facilitating better access. The resources available for students seeking academic assistance have been further increased by the advancement of Internet technology.

## 5. Discussion

### 5.1. Purpose and the Main Findings of the Study

The primary purpose of this review was to provide a comprehensive analysis of the behavior of college students seeking academic assistance. Using the PRISMA method, we reviewed the literature and pulled 55 articles from two databases. Two different approaches were used in this paper based on the analysis of these articles. The first section is quantitative, highlighting numerical data taken from ATLAS.ti 22, and it concentrates on the 55 articles’ study year, their country and region of publication, and the journal. The second section is qualitative and investigates the process of AHS behavior, including whom to ask for help, how to ask for help, the outcomes of help-seeking, and the available resources for help-seeking. The study also looks at the increasingly varied ways that students are using the Internet to get academic assistance since the dawn of the Internet era. The anonymity, timeliness, and convenience of the Internet make it a good option for students looking for assistance. The study also revealed that some students require academic assistance but reject it, which necessitates additional study.

### 5.2. Research Implications

Both theoretical and applied implications can be drawn from this study. There are two practical implications of this study. The universities can employ to increase the uptake of academic help systems, such as enhancing outreach and communication efforts, reducing barriers to access, and fostering a supportive and inclusive environment. Second, the students themselves, actively seek academic assistance from peers, teachers, and the university’s academic help resource center when they encounter academic challenges on the university campus, realizing that academic assistance is not a failure and is beneficial for promoting their academic progress.

Theoretically speaking, this study offers a systematic overview of students’ AHS behaviors. For instance, in terms of theoretical implications, we discovered that students’ academic help-seeking behaviors can enhance students’ academic performance. Positive factors like a sense of belonging perceived academic ability and perceived faculty benevolence can encourage students’ behavior of asking for help; negative factors primarily include lower socioeconomic status backgrounds, having a sense of exclusion from classmates, a sense of threat, ambivalence, and the perception that doing so poses a threat can hinder students’ help-seeking behavior. The theoretical contribution primarily aims to increase the body of knowledge about academic help-seeking, spot research holes, and suggest future research directions.

### 5.3. Research Limitations

Like many studies, we acknowledge some limitations to our research. Concerning the limitations of this research, the methodological limitations of the thematic review can be mentioned, such as publication bias [89]. This is because there may be many studies that were not published. After all, they did not reach statistical significance because there was no way to publish them in the databases that were investigated. The retrospective nature of thematic reviews is also a problem in this case. This means that readings of the results of the studies analyzed could be skewed by interpretations that do not make sense in the context of which the studies were conducted.

Moreover, the combination of our keywords may have restricted our search results. In addition, we omitted other research databases, gray literature, book chapters, and reports. These publications may contain additional vital information regarding the AHS behaviors of college students. Moreover, the review process for this study only considered English-language peer-reviewed articles. Therefore, the findings of this study may not be sufficiently exhaustive. College students’ AHS behaviors are still in a concerning stage. Thus, additional journal articles containing empirical findings will continue to emerge. In future studies, more recently published academic help-seeking research should be considered.

### 5.4. Research Contributions

Despite some limitations, this review contributes to the research on college students’ academic help-seeking in several ways. First, this study presents the fundamental characteristics of the studies included, such as sample characteristics, research context, and research countries and published journals. Second, this study summarizes students’ AHS behavior, the relationship between academic help-seeking and academic performance, different sources of AHS, AHS facilitating and hindering factors, and how students seek academic help in the Internet era. Third, the findings of this review provide an empirical landscape of research on students at AHS. Further, this paper suggested four future research directions, which may help researchers identify related topics in this subject area.

### 5.5. Future Studies

**Figure 6:**
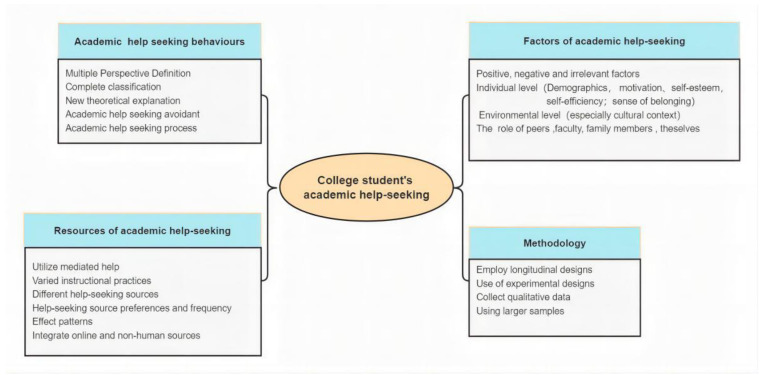
Future research on the existing discussion for college students’ academic help-seeking.

Future research could go in new directions, according to the thematic review. Figure 6 provides 19 research directions to better assist students in utilizing the resources at their disposal to further their academic success by better understanding their behavior when seeking academic assistance. The following categories may be used to classify future research based on the current research scenario and the proposed structure:

Academic help-seeking behaviors: The definition of AHS should be broadened, and students should be considered a significant source of AHS. Future studies ought to investigate the types of assistance offered by faculty and their effects on students’ help-seeking behavior. Concurrently, it is necessary to investigate new models of learning behavior and AHS behavior.

Resources for academic help-seeking: How do they choose between formal and informal resources? How do they decide between face-to-face and online help-seeking methods? Future studies should create a networked system of student help-seeking behaviors and offer a more thorough understanding of the help-seeking environment. To help students use a variety of AHS methods to achieve better academic performance, future research should also combine online and face-to-face help-seeking.

Factors of academic help-seeking: Future research should also consider the impact of individual characteristics such as age and experience on the decision to seek academic assistance. Additionally, it would be advantageous to conduct additional research on the influence of cultural and socio-economic background on the assistance-seeking behavior of college students. The influence of academic self-efficacy, other people and environments, and different instructor characteristics on students’ help-seeking behavior is an additional avenue worthy of investigation. The influence of comments and interactions on AHS behavior should be considered when engaging in academic help-seeking behavior on social media.

Methodology: Future research should utilize larger samples from a variety of institutions to obtain a comprehensive and unified view of students’ perceptions of academic support services. Researchers should also employ multiple methodologies to investigate the mechanisms of academic help-seeking behavior in greater detail. In future research, meta-analyses could be conducted, such as examining the association between academic help-seeking behavior and academic achievement or investigating the impact of gender and peer assistance on help-seeking behavior. Meta-analysis allows for the synthesis and summarization of findings from literature reviews or reviews, enabling the aggregation and combination of data from multiple independent studies to provide more persuasive and consistent conclusions.

## 6. Conclusions

The main conclusion of this review is that student help-seeking behaviors have been mainly studied from the academic perspective and rarely seen from the perspective of students themselves; When examining help-seeking behaviors, more research that directly incorporates students’ perspectives is needed. This will involve considering the experiences, motivations, and challenges students face when seeking academic support, which may provide valuable insights and a more comprehensive understanding of the topic. In addition, the classification of student help-seeking behaviors has been studied in more detail so far. Most research on academic help-seeking behavior and academic performance is favorable, but different types of academic help-seeking behavior have different effects on academic performance. In addition, current research on the factors influencing students’ help-seeking behaviors has been conducted independently, examining the effects of a single component on academic help-seeking behaviors without considering the effects of multiple factors on academic help-seeking behaviors collectively. Moreover, in the Internet era, students have greater access to online help-seeking resources; however, there is a need for continued research on traditional academic help-seeking behaviors and Internet help-seeking behaviors to facilitate students’ academic progress. And, to develop a strong theoretical body on students’ academic help-seeking behaviors, additional research is required to better understand students’ academic help-seeking behaviors, whether there are differences in help-seeking behaviors across disciplines, ages, and school levels, and which types of academic help-seeking behaviors are more conducive to students’ academic progress.

## Figures and Tables

**Table 1:** Search string.

Search Builder	Search String
Help seeking	Online academic help-seeking OR OAHS OR academic help-seeking OR AHS OR academic help-seeking’ OR ‘academic advising’ OR ‘academic advice’ OR ‘intrusive advising’ OR ‘support service’ OR ‘support services’ OR ‘academic support’ OR “help seeking” OR “help-seeking” OR “Help-seeking behavior” “Help-seeking intentions” OR “Learning strategies” OR “Question asking” OR “self-regulated learning”
College students	“university” OR “college” OR “academy” OR “higher education” “university student” OR “university students” OR “college student” OR “college students”

**Table 2:** The number of articles published in the journal.

	2012	2013	2014	2015	2016	2017	2018	2019	2020	2021	2022	Totals
Frontiers in Education	-	-	-	-	-	-	-	1	-	2	1	4
Internet and higher education	-	1	1	-	-	-	1	-	-	-	-	3
Journal of academic librarianship	-	-	1	-	1	1	-	-	-	-	-	3
International journal of educational technology in higher education	-	-	-	-	-	-	1	-	-	1	-	2
Journal of college student development	-	-	1	-	-	-	-	1	-	-	-	2
TechTrends	-	-	-	-	-	1	-	-	-	1	-	2
Active learning in higher education	-	-	-	-	-	-	-	-	-	1	-	1
American journal of pharmaceutical education	-	1	-	-	-	-	-	-	-	-	-	1
Australasian journal of engineering education	-	-	-	-	-	-	1	-	-	-	-	1
Bangladesh journal of medical science	-	-	-	-	-	-	-	-	1	-	-	1
Bmc medical education	-	-	-	-	-	-	-	-	1	-	-	1
British journal of educational psychology	-	-	-	1	-	-	-	-	-	-	-	1
Computers and education	-	-	-	-	-	-	1	-	-	-	-	1
Computers in human behavior	-	-	-	-	1	-	-	-	-	-	-	1
Cultural Diversity\and ethnic minority psychology	-	-	-	-	-	-	-	-	1	-	-	1
Cypriot journal of educational sciences	-	-	-	-	-	-	-	-	1	-	-	1
Distance education	-	-	1	-	-	-	-	-	-	-	-	1
E-learning and digital media	1	-	-	-	-	-	-	-	-	-	-	1
Education and information technologies	-	-	-	-	-	-	-	-	-	1	-	1
Educational psychology	1	-	-	-	-	-	-	-	-	-	-	1
Electronic journal of research in educational psychology	-	-	-	-	-	-	-	-	-	1	-	1
Enfermeria clinica	-	-	-	-	-	-	-	-	1	-	-	1
European journal of psychology of education	-	-	-	-	-	-	-	-	-	-	1	1
Healthcare (Switzerland)	-	-	-	-	-	-	-	-	-	-	1	1
High ability studies	-	-	-	-	-	-	-	1	-	-	-	1
Information research-an international electronic journal	-	-	-	-	-	-	-	1	-	-	-	1
Journal of applied developmental psychology	-	-	-	-	-	-	-	-	1	-	-	1
Journal of career development	-	-	-	-	-	1	-	-	-	-	-	1
Journal of chemical education	-	-	-	-	-	-	-	-	-	1	-	1
Journal of computer assisted learning	-	-	1	-	-	-	-	-	-	-	-	1
Journal of computing in higher education	-	-	-	-	-	-	-	-	-	1	-	1
Journal of diversity in higher education	-	-	-	-	-	-	-	-	-	1	-	1
Journal of experimental education	-	-	-	-	-	-	-	-	-	1	-	1
Journal of further and higher education	-	-	-	-	-	-	-	-	1	-	-	1
Learning and individual differences	-	-	-	-	-	-	1	-	-	-	-	1
Masculinities and social change	1	-	-	-	-	-	-	-	-	-	-	1
Personal relationships	-	-	1	-	-	-	-	-	-	-	-	1
Research in higher education	-	-	1	-	-	-	-	-	-	-	-	1
Social psychology of education	-	1	-	-	-	-	-	-	-	-	-	1
Teaching and learning inquiry	-	-	-	-	-	-	-	-	-	-	1	1
Thinking and reasoning	-	-	-	-	-	-	-	-	1	-	-	1
Universal access in the information society	-	-	-	-	-	1	-	-	-	-	-	1
Urban education	-	-	-	-	-	-	-	1	-	-	-	1
Urban review	-	-	-	-	-	-	-	-	-	1	-	1
Zeitschrift fur entwicklungspsychologie und padagogische psychologie	-	-	-	-	-	-	-	-	-	1	-	1
Total	3	3	7	1	2	4	5	5	8	13	4	55

## Data Availability

Not applicable.

## Supplementary Materials

The following supporting information can be downloaded at: https://www.mdpi.com/article/10.3390/bs13080637/s1.

## Appendix A

**Table A1:** Crowe Critical Appraisal Tool (CCAT) form.

Crowe Critical Appraisal Tool (CCAT) form			
Category Item	Item Descriptors	Description	Score (1–5)
1. Preliminaries			
Title	1. Includes study aims and designs		
Abstract	1. Key information 2. Balanced and informative		
Last	1. Sufficient detail others could reproduce 2. Clear/concise writing, table(s), diagram(s), and figure(s)		
		Preliminaries (/5)	
2. Introduction			
Background	1. Summary of current knowledge 2. Specific problem(s) addressed and reason(s) for addressing		
Objective	1. Primary objective(s), hypothesis(es), or aim(s) 2. Secondary question(s)		
Is it worth continuing?		Introduction (/5)	
3. Design			
Research design	1. Research design was chosen and why 2. Suitability of research design(s)		
Intervention, treatment, exposure	1. Intervention(s)/treatment(s)/exposure(s) chosen and why 2. Precise details of intervention(s)/treatment(s)/exposure(s) for each group 3. Intervention(s)/treatment(s)/exposure(s) valid and reliable		
The outcome, output, predictor, measure	1. Outcome(s)/output(s)/predictor(s)/measure(s) chosen and why 2. Clearly define outcome(s)/output(s)/predictor(s)/measure(s) 3. Outcome(s)/output(s)/predictor(s)/measure(s) valid and reliable		
Bias, etc.	1. Potential bias, confounding variables, effect modifiers, interactions 2. Sequence generation, group allocation, group balance, and by whom 3. Equivalent treatment of participants/cases/groups		
Is it worth continuing?		Design (/5)	
4. Sampling			
Sampling method	1. Sampling method(s) chosen and why 2. Suitability of sampling method		
Sampling size	1. Sampling size, how chosen, and why 2. Suitability of sample size		
Sampling protocol	1. Target/actual/sample population(s): description and suitability 2. Participants/cases/groups: inclusion and exclusion criteria 3. Recruitment of participants/cases/groups		
Is it worth continuing?		Sampling (/5)	
5. Data collection			
Collection method	1. Collection method(s) chosen and why 2. Suitability of collection method(s)		
Collection protocol	1. Include date(s), location(s), setting(s), personnel, material(s), and process(es) 2. Methods to ensure/enhance the quality of measurement/instrumentation 3. Manage non-participation, withdrawal, incomplete/lost data		
Is it worth continuing?		Data collection (/5)	
6. Ethical matters			
Participant ethics	1. Informed consent, equity 2. Privacy and confidentiality/anonymity		
Researcher ethics	1. Ethical approval, funding, conflict(s) of interest 2. Subjectivities and relationship(s) with participants/cases		
Is it worth continuing?		Ethical matters (/5)	
7. Results			
Analysis, Integration, Interpretation method	1. A.I.I. method(s) for primary outcome(s)/output(s)/predictor(s) chosen and why 2. Additional A.I.I. methods (e.g., subgroup analysis) chosen and why 3. Suitability of analysis/integration/interpretation method		
Essential analysis	1. Flow of participants/cases/groups through each stage of research 2. Demographic and other characteristics of participants/cases/groups 3. Analyze raw data, response rate, non-participation/withdrawal/incomplete/lost data		
The outcome, output, predictor analysis	1. Summary of results and precision for each outcome output/predictor/measure 2. Consideration of benefits/harms, unexpected results, and problems/failures 3. Description of outlying data (e.g., diverse cases, adverse effects, and minor themes)		
		Results (/5)	
8. Discussion			
Interpretation	1. Interpretation of results in the context of current evidence and objectives 2. Draw inferences consistent with the strength of the data 3. Consideration of alternative explanations for observed results 4. Account for bias, confounding/effect modifiers/interactions/ imprecision		
Generalization	1. Consideration of the overall practical usefulness of the study 2. Description of generalizability (external validity) of the study		
Concluding remarks	1. Highlight the study’s particular strength 2. Suggest steps that may improve future results (e.g., limitations) 3. Suggest further studies		
		Discussion (/5)	
9. Total			
Total score	1. Add all scores for categories 1–8		

Note: Scoring for each category is based on the guiding principles recommended in the Crowe Critical. Crowe Critical Appraisal Tool (CCAT): Version 1.4 (19 November 2013): Michael Crowe (michael.crowe@my.jcu.edu.au).

## Appendix B

**Table A2:** The characteristics and CCAT scores of the 55 studies.

No.	Authors (Year) Country	Title	Aim(s)	Participant Characteristics	Research Design	Main Findings	CCAT Scores
1	Algharaibeh [33] (2020) Jordan	Should I ask for help? The role of motivation and help-seeking in students’ academic achievement: A path analysis model	To investigate the relationship between academic motivation, academic help-seeking, and academic achievement	437 university students	Quantitative	Results illustrate the importance of academic motivation and help-seeking to provide a fuller understanding of students’ academic achievement	36
2	Almaghaslah [9] (2022) Saudi Arabia	Academic help-seeking behaviors of undergraduate pharmacy students in Saudi Arabia: Usage and helpfulness of resources	To explore the reasons behind academic help-seeking, assessing the resources available to assist students, the frequency of use, perceived usefulness, and factors considered when choosing a certain resource	103 students	Quantitative	Results indicated that useful resources like professors and textbooks were consulted less frequently, while frequently used resources like summaries were not particularly helpful, according to the results	40
3	Amador [90] (2014) United States	Academic advising via Facebook: Examining student help-seeking	To focus on how six university students used and understood an electronic social network to seek help from an academic advisor	6 selected participants, all between the ages of 19 and 22.	Qualitative	Results indicated that participants used a social network site to seek academic advising help and considered it beneficial for interacting with higher education personnel electronically	35
4	Amador [15] (2017) United States	Academic help seeking: A framework for conceptualizing Facebook use for higher education support	To understand how higher education students, specifically preservice teachers, used Facebook to seek academic help	Six participants, four females, and two males ranged in age from 19 to 22, with half being first-generation college students	Qualitative	Results indicated that regular use of Facebook for academic support created a social network that fostered a sense of community and helped with academic tasks	32
5	Aristoteles [63] (2020) Republic of Indonesia	The relationship of academic help-seeking with student achievement in nursing students in Stokes Muhammadiyah Palembang	To determine the relationship of academic help-seeking with student achievement in the Nursing Study Program Stokes Muhammadiyah Palembang	116 respondents	Quantitative	Results indicated that there is a relationship between academic help-seeking and student achievement in Nursing Science Studies	39
6	Beisler [55] (2016) United States	How do students get help with research assignments? Using drawings to Understand Students’ Help-Seeking Behavior	To explore undergraduate students’ help-seeking behavior about writing papers that require research	220 undergraduate students	Quantitative	Results indicated that students usually receive help from peers and family members after drafting papers. Research-related tasks are the most challenging, but librarians are rarely sought for help	37
7	Bizuneh [91] (2022) Ethiopia	Belief in counseling service effectiveness and academic self-concept as correlates of academic help-seeking behavior among college students	To examine the association between homework practices of college students, motivation, self-regulation of learning, and final course grades	133 college students drawn from a small private college in urban New York	Quantitative	Results indicated that using homework logs and considering self-efficacy, motivation, and help-seeking strategies related to homework completion can promote self-directed learning	30
8	Bornschlegl [92] (2021) Australia	Application of the theory of Planned Behavior to identify variables related to academic help seeking in higher education	To identify personality variables, background variables, and variables related to the Theory of Planned Behavior that can predict academic help-seeking in higher education to inform the design of engaging and accessible academic support	430 students participated in the survey	Quantitative	The study found that only a small portion of the variance of academic help-seeking behavior could be explained, indicating the need for interventions to increase help-seeking	39
9	Bornschlegl [93] (2022) Australia	Increasing accessibility to academic support in higher education for diverse student cohorts	To clarify why students (do not) engage in support and what could be changed to make services more accessible and engaging	174 students	Quantitative	Results indicated that improve promotion and address the stigma around seeking help to normalize accessing academic support. Offer various modes of support to increase helpfulness and positive perception of services	37
10	Broadbent [84] (2021) Australia	Use of live chat in higher education to support self-regulated help-seeking behaviors: a comparison of online and blended learner perspectives	To explore students’ perceptions of the use of live chat technology for online academic help-seeking within higher education, with a focus on comparing online and blended learners’ perspectives	246 students who were studying psychology online (n = 91) or in blended learning (n = 155) environments	Quantitative	Results indicated that live chat technology was well-received by both groups, with online learners reporting greater satisfaction, access to staff, and feeling cared for by the teaching team	38
11	Brouwer [76] (2022) Netherlands	The role of prosocial attitudes and academic achievement in peer networks in higher education	To investigate to what extent students’ prosocial attitudes and academic achievement facilitate the embeddedness in friendship and help-seeking networks while taking structural network characteristics into account	95 first-year bachelor’s degree students	Quantitative	Results indicated that students’ prosocial attitudes and achievement influenced friendship formation, while only their achievement affected the formation of help-seeking relationships	40
12	Brown [94] (2020) UK	Barriers to academic help-seeking: the relationship with gender-typed attitudes	To investigate the relationship between gender-typical attitudes and reluctance to seek academic help	162 students at six UK universities	Quantitative	Results indicated that reluctance to seek help was predicted by higher scores on the masculine gender script subscale Mastery and Control of feelings for both male and female students	38
13	Chang [49] (2020) United States	The complexity of cultural mismatch in higher education: norms affecting first-generation college students’ coping and help-seeking behaviors	To explore how cultural norms affect coping and help-seeking for academic, financial, and psychological problems among diverse first-generation college students	11 individual interviews	Qualitative	Results indicated that first-generation college students face a mismatch between hard and soft independence, hindering their help-seeking behavior due to relational concerns	40
14	Cheng [95] (2013) Republic of China (Taiwan)	University students’ online academic help seeking: The role of self-regulation and information commitments	To investigate if students’ online academic help-seeking (OAHS) can be facilitated by the aid of technology, and if improvement in OAHS involves personal variables such as self-regulated learning (SRL), and ‘information commitments’ (ICs), which are evaluative standards and strategies of online information	328 university students in Taiwan.	Quantitative	The results verify that the students’ perceived SRL mediates the relationships between their perceptions of their ICs and OAHS to some degree	37
15	Dunn [74] (2014) United States	Influence of academic self-regulation, critical thinking, and age on online graduate students’ academic help-seeking	To explore the effect of academic self-regulation, critical thinking, and age on online graduate students’ help-seeking	165 graduate students	Quantitative	Results indicate that these variables did significantly influence help-seeking and that as self-regulation and critical thinking increased so did help-seeking. However, as age increased, help-seeking decreased	33
16	Ellis [96] (2021) United States	A Theory of Reasoned Action Approach to examining academic help-seeking behaviors among adolescents in a college readiness program	To examine the extent to which GEAR UP participant attitudes, subjective norms, and intentions influence whether participants sought academic support from teachers, counselors, parents, or friends during an academic semester	67 students	Quantitative	Results indicated that GEAR UP students are influenced by subjective norms when seeking academic support from friends, but less so when seeking support from school personnel or parents	37
17	Finney [51] (2018) United States	Exploring profiles of academic help seeking: A mixture modeling approach	To explore the most common profiles of academic help-seeking in college student populations. Specifically, mixture modeling was used to identify and evaluate naturally occurring combinations, or patterns, of help-seeking attitudes and behaviors	1950 incoming first-year college students	Quantitative	Results indicated that person-centered techniques revealed typical profiles of complex help-seeking processes among upper-class and first-year students, with one distinct profile. Implications are discussed	35
18	Giblin [97] (2021) United States	Examining decision-making processes and heuristics in academic help-seeking and instructional environments	To explore how college students describe their decision-making process regarding the selection of help-seeking sources	25 undergraduate students	Qualitative	Results indicated that the examination of decision-making heuristics may provide a new method to explore help-seeking behavior	40
19	Giblin [61] (2021) United States	An exploration of factors influencing the decision-making process and selection of academic help sources	To identify factors that influence undergraduate students’ selection of a source of help	99 math class college students	Quantitative	Findings support adding online sources to help-seeking models and highlight the importance of relationships. A new theory of source selection emerged, integrating help-seeking and information-searching behavior	40
20	Hao [82] (2016) United States	What is the most important prediction of computer science students’ online help-seeking behaviors	To investigate the most important predictors of computer science students’ online help-seeking behaviors	Two groups of 203 undergraduate students	Quantitative	Results indicated that problem difficulty was the most important predictor for all three types of online help-seeking, followed by learning proficiency level, academic performance, and epistemological belief	38
21	Hayman [86] (2019) Canada	Information behavior of undergraduate students using Facebook Confessions for educational purposes	To investigate the information behavior of undergraduate students seeking academic help via anonymous posts to a university Facebook Confessions page. While Confessions pages have gained popularity in post-secondary contexts, their use for educational purposes is largely unexplored	2712 confessions were posted during one academic year.	Quantitative	Results indicated that Facebook Confessions is used for academic support through help-seeking, advice-giving, information-seeking, and moderating behaviors, based on qualitative and quantitative analysis	39
22	Holt [98] (2014) United States	Attitudes about help-seeking mediate the relation between parent attachment and academic adjustment in first-year college students	To examine first-year college students’ attitudes about academic help-seeking as one possible mechanism	93 first-year students	Quantitative	Results indicated that help-seeking attitudes mediated the relationship between parent attachment and academic adjustment, with females having more positive attitudes towards help-seeking, according to results	33
23	Holt [99] (2014) United States	Help-seeking and social competence mediate the parental attachment–college student adjustment relation	To investigate whether attitudes about academic help-seeking, social competence, and self-compassion mediated the relations between parental attachment and college student adjustment	204 first-year students	Quantitative	Results suggested that help-seeking attitudes and social competence could be fruitful targets of intervention for personnel working with college students who have strained parental relationships	37
24	Johnson [100] (2019) United States	Examining the academic advising experiences of black males at an urban university: An exploratory case study	To understand the academic advising experiences of Black males at a large urban, predominantly White institution	9 Black male college students	Qualitative	Results indicated that participants experienced process-related challenges and noted the impact of race/culture in academic advising but found positive outcomes in both formal and informal advising	37
25	Long [79] (2018) United States	Self-compassion is associated with reduced self-presentation concerns and increased student communication behavior	To investigate whether students’ levels of self-compassion (the tendency to be mindful and kind to oneself and to recognize one’s common humanity) would be associated with a lower fear of evaluation and higher academic communication behavior	691 undergraduates	Quantitative	Results indicated that students with higher self-compassion exhibited lower classroom participation avoidance and reported a higher tendency to ask questions, seek help, and speak with their instructors outside	37
26	Mahasneh [59] (2012) Jordan	Academic help-seeking in online and face-to-face learning environments	To compare actual help-seeking frequencies across online and face-to-face learning environments. It also examines strategies enacted by nursing students when they faced academic difficulties, reasons for help-seeking avoidance, and the relationship between the frequency of asking questions and achievement	nursing students: a total of 56 online (n = 25) face-to-face (n = 31).	Quantitative	Results indicated that the desire for autonomy was one of the main reasons for avoiding seeking help. It was also expected that students’ achievement would be significantly correlated with help-seeking frequency	37
27	Marrs (2012) [73] United States	Gender, masculinity, femininity, and help-seeking in college	To explore the possible impact of gender-related attributes such as masculinity and femininity on academic help-seeking behaviors and academic performance	567 college undergraduates	Quantitative	These results highlight the importance of exploring the potential influence of gender-related constructs on academic behavior and performance	36
28	Micari (2021) [32] United States	Is it OK to ask? The impact of instructor openness to questions on student help-seeking and academic outcomes	To examine the relationships among instructor openness to student questions, student help-seeking behavior, and student final grade in lecture-style college/university courses	268 university students	Quantitative	Results indicated that perceived instructor openness and help-seeking were positively related to grades. Help-seeking mediated the relationship between perceived instructor openness to questions and final grade	35
29	Opdecam [77] (2014) Belgium	Preferences for team learning and lecture-based learning among first-year undergraduate accounting students	The first objective of this study is to investigate students’ preferences about their gender, ability, motivation, and learning strategy. The second objective is to explore whether a team-based approach is more effective than lecture-based learning when students participate in their preferred method	291 students	Quasi-experiment	Results indicated that students with a preference for team learning had a lower ability level were more intrinsically motivated, had less control of their learning beliefs, were more help-seeking, and were more willing to share their knowledge with peers	35
30	Parnes [101] (2020) United States	Closing the college achievement gap: Impacts and processes of a help-seeking intervention	To examine how a 4-session, group-based intervention (Connected Scholars) may improve underrepresented student outcomes by increasing help-seeking and network orientation	396 public university students, 65% female, 90% racial/ethnic minority, and 42% first-generation college students (FGCS).	Quantitative	Results indicated that the Connected Scholars program had a positive impact on first-generation college students’ GPA and student–instructor relationships, with changes in help-seeking behavior and network orientation playing a role	40
31	Payakachat [50] (2013) United States	Academic help-seeking behavior among student Pharmacists	To identify factors associated with academic help-seeking behavior among student pharmacists at a public university	Phrase 1 included 6 student pharmacists (1 male and 5 female) In phase 2, 304 out of 443 students (68.6% response rate)	Mixed	Results indicated that academic help-seeking behavior was positively related to greater perceived academic competence and positive relationships among student pharmacists and faculty members	32
32	Payne (2021) [56] United States	“Just because I am first gen doesn’t mean I’m not asking for help”: A thematic analysis of first-generation college students’ academic help-seeking behaviors	To investigate what first-generation students do when they need academic help and (b) how first-generation students navigate and negotiate their resources	17 self-identified first-generation college students	Qualitative	Results indicated that first-generation students engage effectively in help-seeking, utilizing networks to assess and correct help quality, risks, and strategies	40
33	Qayyum [23] (2018) United States	Student help-seeking attitudes and behaviors in a digital era	To examine college students’ attitudes and habits for seeking academic help. Students’ preferences for seeking academic help via digital and non-digital technologies are identified (N = 438)	438 students	Quantitative	Results indicated that perceived threat, perception of instructors, and students’ preference to work independently are significant in predicting whether students sought help from instructors outside of class	37
34	Reeves [57] (2015) United States	A comparison of technology ally mediated and face-to-face help-seeking sources	To examine whether students prefer and intend to utilize technologically mediated or face-to-face help-seeking sources	226 Participants	Quantitative	Results indicated that higher-performing students prefer in-person help while lower-performing students prefer technological means, but all students intended to seek help	36
35	Sakiz [102] (2012) Turkey	Perceived instructor effective support about academic emotions and motivation in college	To examine the associations among perceived instructor affective support, academic enjoyment, academic hopelessness, behavioral engagement, and academic help-seeking in college classrooms	277 college students enrolled in a teacher training department of a major university in Turkey	Quantitative	The study emphasizes the need for supportive learning environments in K12 and college classrooms and discusses the implications for practice and future research, along with limitations	37
36	Santos [81] (2020) France	Do learners decline to seek help to conform to rational principles	Explore why do learners fail to seek help, when doing so would be beneficial	65 students	Experimental study	Results indicated that participants were more likely to seek help when help came at no financial cost but showed little sensitivity to other parameters	36
37	Schlusche [53] (2021) Germany	Perceived social resources affect help-seeking and academic outcomes in the initial phase of undergraduate studies	To investigate the role of peer students as a social resource for academic help-seeking to overcome knowledge-related difficulties	First-semester students: total129 science (n 49) engineering (n 80)	Quantitative	Results show that social variables such as social embeddedness and group awareness can influence academic help-seeking behavior and student success in different study programs	38
38	Schworm [58] (2012) Germany	E-learning in universities: Supporting help-seeking pro-cusses by instructional prompts	To investigate the effect of giving prompts on the quantity and quality of academic help-seeking was experimentally investigated in a blended university learning course of educational science	39 students	Quantitative	Results indicated that students with prompts about help-seeking relevance had better learning outcomes, higher participation, and initiated more discussions	34
39	Schworm [62] (2014) Germany	Learning with video-based examples—are you sure you do not need help	To investigate help-seeking activities in a computer-based environment teaching argumentative skills through videos of argumentative dialogues of teachers who discussed controversial issues in the context of a workshop	43 students	Quantitative	Results revealed the relevance of learners’ response certitude concerning their help use. Low response certitude about the correctness of a task solution led to higher help use which was positively related to the learning outcome	31
40	Shi [103] (2021) China	Characterizing academic help-seeking moods for enrollment performance of institutional online student	To understand learners and associate their performances via exploiting academic help-seeking moods with online learning in institutional education settings	2685 undergraduate courses	Quantitative	Results propose a novel research model and identify three different online help-seeking moods, which are namely goal-directed seeker, exploratory seeker, and avoidant seeker	37
41	Shively [104] (2013) United States	Longitudinal changes in college math students’ implicit theories of intelligence	To examine changes over time in implicit theories of intelligence and their relationships to help-seeking and academic performance	243 students who completed at least one questionnaire 98 students (44 male, 54 female) completed a questionnaire only at the beginning of the semester 84 students (44 male, 40 female) completed a questionnaire only at the end of the semester 61 students (20 male, 41 female) provided data at both waves	Quantitative	Results indicated that students had more incremental views of general than math intelligence. Further, their views became less incremental over the semester; however, this decrease was greater for math than for general intelligence	38
42	Sun [64] (2018) Denmark	The role of self-regulated learning in students’ success in flipped undergraduate math courses	To examine the relationships between academic achievement and three key self-regulatory constructs—prior domain knowledge, self-efficacy, and the use of learning strategies—in two flipped undergraduate math courses	151 undergraduate students from 16 flipped sections of Calculus I and II courses in a large Midwestern university	Quantitative	Results indicated that students’ self-efficacy in learning math and the use of help-seeking strategies were all significantly positively related to academic achievement in both pre-and in-class learning environments	39
43	Tang [105] (2014) United States	Distance students’ attitude toward library help-seeking	To explore library help-seeking attitudes of distance education students, with a particular focus on the stage of identifying helper(s)	220 students enrolled in one or more distance education courses for the Fall term of 2012	Quantitative	Results indicated that distance students who frequently visit the library and seek help have higher self-efficacy. Li guides are the most used source. Near-campus students prefer face-to-face consultation, while far-campus students tend to seek help from a distance librarian. Email is the most common way of distributing and receiving library information	30
44	Thomas [69] (2017) United States	Where students start and what they do when they get stuck: A qualitative inquiry into academic information-seeking and help-seeking practices	To investigate two questions key to academic library resources and services: Which sources are students most likely to use to begin their academic work? Whom do students tend to consult for research assistance?	15 undergraduate and graduate students	Qualitative	Results indicated that students tend to seek help from faculty and peers and use library databases for research but are unaware of librarians’ roles. Small study groups and alternate research sites also emerged as themes	34
45	Thomas [72] (2019) United States	Predicting Academic Help-Seeking Intentions Using the Reasoned Action Model	To use the Reasoned Action Model to investigate the determinants of students’ intentions to utilize university-based sources of academic support	Participants (N = 125) in Study 1 Participants (N = 176) in Study 2	Qualitative	Results indicated that normative pressure was the strongest predictor of intentions to use university-based academic support, followed by attitudes. These results suggest that interventions targeting normative and behavioral beliefs may be effective in increasing academic help-seeking	38
46	Tsai [85] (2017) Republic of China (Taiwan)	How to solve students’ problems in a flipped classroom: a quasi-experimental approach	To improve students’ learning, the authors designed and provided flipped learning (FL) and treatment of online academic help-seeking (OAHS) in a computing course to help students develop computing skills for using Microsoft Word and PowerPoint	126 undergraduates	Quantitative	Results indicated that OAHS treatment improved computing skills for Word and PowerPoint, while FL treatment did not show significant improvement	36
47	Umarani [30] (2020) India	Do the Students have the attitude to seek academic help?—A study among undergraduate students	To assess the attitude on academic help-seeking behavior among nursing students	Among 96 first-year B.Sc. Nursing students	Quantitative	Results indicated that students have a positive attitude towards academic help-seeking, and educators should address the factors that hinder it. Help-seeking is a useful strategy that benefits academic success	32
48	Valenzuela [106] (2021) Chile	Variables Predicting Participation in institutional academic support services (IAS)	To identify the variables that best predict voluntary participation in these services	803 male and female university students from four Chilean universities participated in this study	Quantitative	Results indicated that predictors of participation in institutional academic support services include awareness of need, knowledge of procedures, experience seeking help, and cost associated with loss of alternatives	38
49	Vilkova [88] (2021) Russia	Deconstructing self-regulated learning in MOOCs: In search of help-seeking mechanisms	To fill the gap in understanding the structure of SRL skills utilizing the Online Self-Regulated Learning Questionnaire (OSLQ)	913 Russian MOOC learners	Quantitative	Results indicated that the dimension ‘help-seeking’ is not effective in the MOOC environment	37
50	Williams-Dobosz [107] (2021) United States	Ask for help: Online help-seeking and help-giving as indicators of cognitive and social presence for students underrepresented in chemistry	To analyze help-seeking behaviors and responses to requests for help in an online college-level chemistry course’s discussion forum	94 students enrolled in and completed an online, asynchronous, early curriculum, college-level chemistry course	Quantitative	Results indicated that requests for help were responded to equally, regardless of how explicitly students appealed for help	36
51	Wirtz [71] (2018) United States	Resource usage and usefulness: academic help- seeking behaviors of undergraduate engineering students	To explore the Help-seeking behaviors of students enrolled in a Mechanical Engineering program at a large research-intensive university	355 survey respondents 14 individual conversations and 23 group conversations consisting of 92 participants	Mixed	Results indicated that the more convenient a resource is perceived to be, the more likely a student is to use that resource	35
52	Won [78] (2021) United States	Brief research report: Sense of Belonging and academic help-seeking Self-regulated learning	To investigate whether college students’ sense of belonging could be used to understand their academic help-seeking	307 College students	Quantitative	Results indicated that a sense of belonging predicted adaptive help-seeking, along with self-efficacy for self-regulated learning, while utility value predicted expedient help-seeking. Results can inform interventions to promote effective help-seeking	37
53	Xie [108] (2019) China	Effects of undergraduates’ academic self-efficacy on their academic help-seeking behaviors: The mediating effect of professional commitment and the moderating effect of gender	To examine whether gender plays a moderating role in the association between academic self-efficacy and academic help-seeking	559 university students	Quantitative	Findings show that greater professional commitment is associated with more instrumental academic help-seeking and less avoidance of help-seeking	34
54	Zander [52] (2021) Germany	Perceived peer exclusion as a predictor of students’ help-seeking strategies in higher education	To investigate the relationship between perceived peer exclusion and help-seeking strategies	447 undergraduate students in 25 seminars and tutorials	Quantitative	Results indicated that help-seeking in computer science signals competence-related inferiority, which indicates an “image problem.” Enhancing adaptive help exchange cultures is necessary to address this issue	37
55	Zheng [65] (2020) China	Self-regulated learning: the effect on medical student learning outcomes in a flipped classroom environment	To explore which self-regulated learning skills affect student learning performance in the first 2 years of medical school at a university in the midwestern United States	146 first- and second-year medical students	Quantitative	Results indicated that the use of peer learning and help-seeking positively affected the performance of first- and second-year students, respectively, whereas the use of rehearsal harmed student learning outcomes	36
